# Inzidenz des plötzlichen Epilepsietodes (SUDEP): Update und Limitationen

**DOI:** 10.1007/s00115-023-01595-0

**Published:** 2024-01-22

**Authors:** Hannes Wartmann, Timo Effenberger, Hendrik Klähn, Timm Volmer, Rainer Surges

**Affiliations:** 1 SmartStep Data Institute GmbH, Hamburg, Deutschland; 2 Klinik und Poliklinik für Epileptologie, Universitätsklinikum Bonn, Venusberg-Campus 1, 53127 Bonn, Deutschland

**Keywords:** Plötzlicher, unerwarteter Tod bei Epilepsie, Vorzeitige Mortalität, Kardiorespiratorisches Versagen, Epilepsie, Tonisch-klonische Anfälle, Sudden unexpected death in epilepsy, Premature mortality, Cardiorespiratory failure, Epilepsy, Tonic-clonic seizures

## Abstract

**Hintergrund:**

Der plötzliche, unerwartete Tod bei Epilepsie (SUDEP) ist in den meisten Fällen wahrscheinlich eine fatale Komplikation tonisch-klonischer Anfälle und trägt maßgeblich zur vorzeitigen Sterblichkeit von Menschen mit Epilepsie bei. Die Angaben zum SUDEP-Risiko schwanken erheblich je nach Studienpopulation, sodass systematische Zusammenfassungen unter Einbeziehung aktueller Studien zur belastbaren Schätzung und Verbesserung der Beratung von Menschen mit Epilepsie erforderlich sind.

**Ziel der Arbeit:**

Ziel der Arbeit ist es, einen Überblick über die gegenwärtige Forschungslage hinsichtlich der SUDEP-Inzidenz in unterschiedlichen Patientenpopulationen zu geben und potenzielle Schlussfolgerungen sowie bestehende Limitationen zu diskutieren.

**Material und Methoden:**

Es wurde eine systematische Literaturrecherche zur Inzidenz des SUDEP in MEDLINE und EMBASE mit ergänzender Handsuche im Juni 2023 durchgeführt. Von insgesamt 3324 Publikationen wurden 50 für diese Arbeit ausgewertet.

**Ergebnisse:**

Die analysierten Studien zeigen eine signifikante Heterogenität in Bezug auf Kohorten, Studiendesign und Datenquellen. Untersuchungen, die ohne spezifische Kriterien durchgeführt wurden und sich auf umfangreiche Register stützten, weisen eine Inzidenz von 0,78 bis 1,2 pro 1000 Patientenjahre auf. Studien, welche die Inzidenz für mehrere Altersgruppen angeben, weisen mehrheitlich eine Erhöhung der Inzidenz mit zunehmendem Alter auf; mit dem Höhepunkt im mittleren Alter.

**Diskussion:**

Aufgrund unterschiedlicher Methoden der Datenerhebung und Inzidenzberechnung gestaltet sich ein Vergleich zwischen den Studien als herausfordernd. Die Verbindung zwischen Lebensalter könnte möglicherweise auf eine Unterrepräsentation von Kindern, Jugendlichen und Patienten über 60 Jahre zurückzuführen sein.

**Schlussfolgerung:**

Betrachtet man alle Altersgruppen und Epilepsieformen, ergibt sich, dass jährlich etwa 1 von 1000 Menschen mit Epilepsie an SUDEP verstirbt. Bei einer angenommenen Epilepsieprävalenz von 0,6 % in Deutschland könnte dies zu mehr als einem SUDEP-Fall täglich führen. Um zu weiteren Erkenntnissen zu gelangen, ist eine Standardisierung der Untersuchungsmethoden essenziell.

## Abstract (en)

**Background:**

Sudden unexpected death in epilepsy (SUDEP) is in most cases probably due to a fatal complication of tonic-clonic seizures and plays a significant role in the premature mortality of individuals with epilepsy. The reported risks of SUDEP vary considerably depending on the study population, so that an up-dated systematic review of SUDEP incidence including most recent studies is required to improve the estimated SUDEP risk and the counseling of individuals with epilepsy.

**Objective:**

To provide an overview of the current research landscape concerning SUDEP incidence across different patient populations and discuss potential conclusions and existing limitations.

**Material and methods:**

A systematic literature review on SUDEP incidence was conducted in MEDLINE and EMBASE, supplemented by a manual search in June 2023. Out of a total of 3324 publications, 50 were reviewed for this study.

**Results:**

The analyzed studies showed significant heterogeneity concerning cohorts, study design and data sources. Studies conducted without specific criteria and relying on comprehensive registers indicated an incidence of 0.78–1.2 per 1000 patient-years. Research providing incidences across various age groups predominantly show an increase with age, peaking in middle age.

**Discussion:**

Due to varying methods of data collection and incidence calculation, comparing between studies is challenging. The association with age might be due to an underrepresentation of children, adolescents and patients over 60 years.

**Conclusion:**

Considering all age groups and types of epilepsy it is estimated that about 1 in 1000 individuals with epilepsy dies of SUDEP annually. With an assumed epilepsy prevalence of 0.6% in Germany, this could lead to more than one SUDEP case daily. Standardization of research methods is essential to gain more profound insights.

## Infobox SUDEP („sudden unexpected death in epilepsy“)

Definition: SUDEP ist der plötzliche, unerwartete Tod eines Menschen mit Epilepsie, der sich unter gutartigen Bedingungen und ohne ersichtliche Ursache ereignet [36]. Ein „definitiver SUDEP“ wird belegt durch eine Autopsie, bei der alternative Todesursachen ausgeschlossen werden.

Risikofaktoren: Nächtliche Anfälle, allein leben bzw. schlafen und tonisch-klonische Anfälle wurden als stärkste Risikofaktoren identifiziert.

Pathophysiologie: Die Mehrzahl der SUDEP-Fälle wird wahrscheinlich unmittelbar durch einen tonisch-klonischen Anfall ausgelöst, bei dem es nach Anfallsende zunächst zu einem zentralen Atemstillstand kommt, gefolgt von einer schweren Hypoxämie und terminalen Asystolie („fatale SUDEP-Kaskade“). Eine frühe kardiopulmonale Reanimation während des zentralen Atemstillstandes kann wahrscheinlich in vielen Fällen einen SUDEP abwenden und erklärt, warum sich die meisten SUDEP-Fälle unbeobachtet ereignen. Ein sehr viel kleinerer Anteil der SUDEP-Fälle ist auf ventrikuläre Tachyarrhythmien im zeitlichen Zusammenhang mit tonisch-klonischen Anfällen oder ohne direkten Bezug zu epileptischen Anfällen zurückzuführen.

## Hintergrund

In Deutschland sind ausgehend von einer Prävalenz von 0,5–1 % schätzungsweise 650.000 Menschen von einer Epilepsie betroffen [7]. Menschen mit Epilepsie (MmE) haben im Vergleich zur Allgemeinbevölkerung ein erhöhtes Risiko, vorzeitig zu versterben. Das standardisierte Mortalitätsverhältnis liegt dabei zwischen 2 und 2,6 [33, 34, 37]. Hauptursachen für den vorzeitigen Tod sind Lungenentzündungen, Tumoren sowie Herzinfarkte und Schlaganfälle. Weiter kommen tödliche Unfälle, wie beispielsweise durch Ertrinken, und Suizide bei MmE häufiger vor [58]. Besonders relevante, direkt mit der Epilepsie assoziierte Todesursachen sind der Status epilepticus und der plötzliche unerwartete Tod bei Epilepsie, kurz SUDEP („sudden unexpected death in epilepsy“) genannt ([33, 59]; Infobox).

Das Risiko von MmE, plötzlich und unerwartet zu versterben, ist ca. 24-fach erhöht [17]. Die Mehrheit der plötzlichen Todesfälle ist SUDEP zuzuschreiben, welcher bis zu 17 % aller vorzeitigen Todesfälle bei Erwachsenen ausmachen soll [33, 59]. Schwache SUDEP-Risikofaktoren sind männliches Geschlecht und früher Epilepsiebeginn. Starke Faktoren sind schwer behandelbare Epilepsie, nächtliche Anfälle und Alleinleben [57, 61]. Der stärkste Risikofaktor sind tonisch-klonische Anfälle [57]. Diese Anfälle führen zu Bewusstseinsverlust, Körperversteifung und Zuckungen. Ein temporärer Atemstillstand kann folgen, bleibt aber meist folgenlos. SUDEP-Fällen liegt meist ein Atemstillstand nach Anfällen, gefolgt von Hypoxämie und Asystolie, zugrunde [62].

Seit über einem Jahrhundert wird über plötzliche Todesfälle bei MmE berichtet. Jedoch wurden erst 1997 einheitliche SUDEP-Kriterien von Nashef et al. [35] und Annegers et al. [3] definiert. Diese halfen dabei, SUDEP verstärkt in den Fokus der Forschung zu rücken [63, 64]. Seit 10 Jahren existiert eine konsolidierte Fassung der SUDEP-Definition sowie ein erweitertes Klassifikationssystem ([36]; Tab. 1). Dennoch gibt es weiterhin große Unterschiede hinsichtlich der Art der Datenerhebung und der betrachteten Populationen bei Studien zur Inzidenz des SUDEP [47, 63]. Klarheit über die SUDEP-Inzidenz ist aus Sicht der Autoren essenziell, um gezielte Interventionen zu etablieren und vermeidbare Todesfälle zu verhindern [47, 57, 63].

**Tab. 1:** Definition und Klassifikation des SUDEP

*Definition *	
SUDEP ist definiert als plötzlicher, unerwarteter, beobachteter oder unbeobachteter Tod eines Menschen mit Epilepsie mit oder ohne Evidenz eines stattgehabten epileptischen Anfalls, der nicht durch Trauma, Ertrinken, Status epilepticus, Intoxikation oder andere innere Ursachen (durch Autopsie ausgeschlossen) verursacht wurde	
*Klassifikation *	*Voraussetzung*
Definitiv („definite“)	Erfüllung der Definition und Ausschluss anderer Todesursachen durch Autopsie
Wahrscheinlich („probable“)	Erfüllung der Definition ohne durchgeführte Autopsie
Möglich („possible“)	Es kommen auch andere Ursachen für den Tod infrage und es wurde keine Obduktion durchgeführt
Beinahe („near“)	Wiederbelebung nach Atem- und Herz-Kreislauf-Stillstand ohne andere Ursache
Kein („no“)	Eindeutige (andere) Todesursache ist bekannt
Nicht klassifizierbar	Unvollständige Informationen, die keine Klassifikation erlauben

Nashef et al. erweiterten 2012 die Klassifikation um den Term „plus“ (für definitiven, wahrscheinlichen oder beinahen SUDEP), bei dem eine Begleiterkrankung möglicherweise gemeinsam mit der Epilepsie den SUDEP verursacht hat [36]

Diese Arbeit bietet einen Überblick über die SUDEP-Häufigkeit in Epilepsiepopulationen, beleuchtet Studienlimitationen und dokumentiert die Situation in verschiedenen Patientengruppen.

## Methoden

### Datenbankrecherche

Die systematische Literaturrecherche in den Literaturdatenbanken EMBASE und MEDLINE via PubMed wurde zuletzt am 30.06.2023 aktualisiert. Die Suchstrategie umfasste sowohl Freitextbegriffe als auch Schlagwörter (MeSH Terms) und beinhaltete die Suche nach ″SUDEP″, ″Sudden Unexpected Death in Epilepsy″, ″sudden″, ″unexpected″, und ″Death″ im Zusammenhang mit ″Epilepsy″ oder ″Seizures″ oder ″Convulsions″ sowie einen Filter für epidemiologische Studien: ((″Sudden Unexpected Death in Epilepsy″[mh] OR ″Sudden Unexpected Death in Epilepsy″[tiab] OR SUDEP[tiab]) OR ((″Death, Sudden″[mh] OR ((sudden[tiab] OR unexp*[tiab]) AND death[tiab])) AND (″Epilepsy″[mh] OR ″Seizures″[mh] OR epilep*[tiab] OR seizure*[tiab] OR convuls*[tiab]))) AND (″epidemiology″[mh Terms] OR ″epidemiolog*″[tiab] OR ″prevalen*″[tiab] OR ″inciden*″[tiab] OR ″regist*″[tiab] OR ″cohort stud*″[tiab]).

**Abb. 1:**
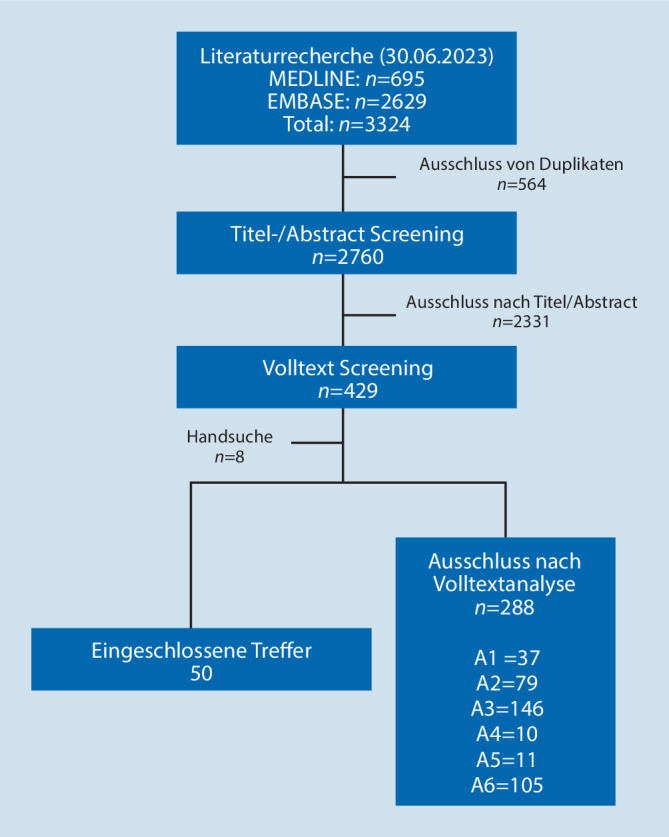
Flussdiagramm ein- und ausgeschlossener Publikationen. *A1* andere Todesursachen, *A2* keine Primärerhebung, *A3* keine Angabe zur Inzidenz, *A4* SUDEP-Definition nicht gemäß Nashef [35] und Annegers [3], *A5* nicht in Deutsch oder Englisch, *A6* keine Vollpublikation

Die Sichtung der Treffer erfolgte durch 2 voneinander unabhängige Reviewer. Eingeschlossen wurden Publikationen ab 1997 (Publikationsjahr der ersten SUDEP-Definition) in deutscher oder englischer Sprache, die Daten zur Inzidenz des SUDEP aus prospektiven oder retrospektiven Studien mit mehr als einem SUDEP-Fall berichten. Systematische Übersichtsarbeiten wurden ebenfalls ausgeschlossen, jedoch wurden die Referenzlisten gesichtet, um die Validität der Suchstrategie zu prüfen und die Vollständigkeit des Studienpools zu gewährleisten (Abb. 1).

### Harmonisierung der Inzidenzen

Inzidenzen werden stets pro 1000 Patientenjahre angegeben. Wenn in einer Publikation keine explizite SUDEP-Inzidenz für 1000 Patientenjahre genannt oder sich nicht ausschließlich auf definitive oder wahrscheinliche Fälle bezogen wurde, wurde die Inzidenz basierend auf der jeweiligen Publikation berechnet (Tab. 2).

**Tab. 2:** Studien mit Bezug auf die SUDEP-Inzidenz in verschiedenen Epilepsiepopulationen

	Land	Datenquelle	Ermittlung der Fälle	SUDEP-Fälle (pro 1000 Patientenjahre)	SUDEP (*n*)	Datensatz (*n*)	Erhebungszeitraum	Alter
*SUDEP bei Kindern und Jugendlichen*								
Ackers (2011) [1]	UK	AED-Studie	KA	0,18^a^	9	6190 MmE	1993–2005	0–18 J
Einarsdottir (2019) [14]	Island	Gerichtsmedizin, AED-Register, Interviews Neurologen	AB, KA	0,2	1	3514 AB	1991–2010	0–14 J
Grønborg (2014) [20]	Dänemark	Epilepsiezentrum	AB, KA, TS	0,8^a^	9	1974 MmE	1999–2008	0–18 J
Holst (2013) [23]	Dänemark	Patientenregister	KA, TS, Todesursachenregister	0,41	50	33.022 MmE	2000–2006	1–35 J
Keller (2018) [28]	Kanada	Pädiatrisches Überwachungsprogramm, pädiatrisches Epilepsie Netzwerk, forensische Pathologie	AB, Int, KA, TS	1,11	16	> 12.000 AB	2014–2015	„Pädiatrische“
Keller (2020) [27]	Kanada	Gerichtsmedizin	AB	0,79	14	15.229 AB	2014–2016	< 16 J
Kløvgaard (2021) [30]	Dänemark	Patientenregister	AB, KA, TS, Todesursachenregister	0,27	k. A.	k. A.	2007–2009	1–17 J
Sveinsson (2017) [60]	Schweden	Patientenregister	AB, KA, TS	1,11	7	57.775 MmE	2008	< 16 J
Weber (2005) [70]	Schweiz	Fallberichte	Fallberichte	0,43	4	k. A.	1984–2001	< 19 J
*SUDEP bei Erwachsenen*								
Karlovich (2020) [26]	USA	Gerichtsmedizin	AB, KA, Tatortuntersuchungen	0,72	44	3732 AB	2014–2017	18–45 J
Keller (2020) [27]	Kanada	Gerichtsmedizin	AB	1,1	128	15.229 AB	2014–2016	> 16 J
Kløvgaard (2021) [30]	Dänemark	Patientenregister	AB, KA, TS, Todesursachenregister	1,21	k. A.	k. A.	2007–2009	18–49 J
Nilsson (1999) [39]	Schweden	Patientenregister	AB, KA, Todesursachenregister	1,5	62	6880 MmE	1980–1989	15–70 J
Wang (2021) [69]	China	Epilepsie Management Programm	Int, TS	1,4	44	10.128 MmE	2010–2019	„Erwachsene“
*SUDEP in der Bevölkerung*								
Aurlien (2012) [5]	Norwegen	Autopsieberichte	AB, KA, Todesursachenregister	0,7	7	384 AB von MmE	1995–2005	16–67 J
Clark (2016) [10]	Australien	Autopsieberichte	AB	0,7	123	537 AB von MmE	2004–2008	k. A.
Einarsdottir (2019) [14]	Island	Gerichtsmedizin, AED-Register, Interviews Neurologen	AB, KA	1,3	37	3514 AB	1991–2010	k. A.
Eslami (2021) [15]	USA	Gerichtsmedizin	AB, KA, Tatortuntersuchungen	0,51	286	k. A.	1983–2018	k. A.
Ge (2017) [18]	China	Populationsstudie	Int, TS	2,03	13	1562 MmE	2010–k. A.	k. A.
Keller (2020) [27]	Kanada	Gerichtsmedizin	AB	1,06	142	15.229 AB	2014–2016	k. A.
Kløvgaard (2021) [30]	Dänemark	Patientenregister	AB, KA, TS, Todesursachenregister	0,99	81	30.437 MmE	2007–2009	1–49 J
Opeskin (2000) [41]	Australien	Gerichtsmedizin	AB	0,33^a^	50	15.751 AB	1991–1997	k. A.
Opeskin (2003) [40]	Australien	Gerichtsmedizin	AB	1,28^a^	50	4375 AB	1997–1999	k. A.
Salmo (2002) [48]	Irland	Pathologie	AB	2,53	22	3103 AB	1991–2000	10–62 J
Sveinsson (2017) [60]	Schweden	Patientenregister	AB, KA, TS	1,2	68	57.775 MmE	2008	k. A.
Tomson (2018) [64]	Schweden	Patientenregister	AB, KA, TS	0,78^a^	235	60.952 MmE	2006–2011	k. A.
*SUDEP an einem Epilepsiezentrum*								
Chamorro-Muñoz (2017/2020) [8]	Spanien	Epilepsiezentrum	AB, KA, TS	0,44	7	2309 MmE	2000–2013	> 13 J
Chen (2005) [9]	Taiwan	Epilepsiezentrum	AB, KA, TS	1,39^a^	3	263 MmE	1991	> 16 J
Duble (2019) [13]	India	Epilepsiezentrum	AB, Int, KA	0,62^a^	4	558 MmE	2000–2004	2–61 J
Khor (2022) [29]	Malaysia	Epilepsiezentrum	Int, KA, TS	0,42	12	2281 MmE	2005–2020	> 14 J
Mohanraj (2006) [33]	Scotland	Epilepsiezentrum	KA, TS, nationales Todesregister	2,11^a^	55	3579 MmE	1981–2001	„Adults“
Papacostas (2015) [42]	Zypern	Epilepsiezentrum	KA	2,13	7	444 MmE	1997–2012	k. A.
Sanchez-Larsen (2019) [49]	Spanien	Epilepsiezentrum	Int, KA	0,96^a^	4	1250 MmE	2010–2018	k. A.
Ryvlin (2013) [45]	Weltweit	Epilepsiezentrum	AB, KA	5,1	16	k. A.	2008–2009	k. A.
Schulz (2019) [50]	Deutschland	Epilepsiezentrum	AB, KA	3,9	14	k. A.	1981–2016	k. A.
Sebera (2020) [51]	Ruanda	Epilepsiezentrum	Int	7,1	3	154 MmE	2016	k. A.
Sillanpää (2013) [54]	Finnland	Epilepsiezentrum	AB, KA	2,65^a^	23	245 MmE	1961–1964	k. A.
Vlooswijk (2007) [66]	Niederlande	Epilepsiezentrum	Int, KA	1,24	29	274 verstorbene Patienten	1999–2004	k. A.
Walczak (2001) [68]	USA	Epilepsiezentrum	AB, Int, KA, TS	1,21	20	4578 MmE	1991–1996	k. A.
*SUDEP bei refraktärer Epilepsie und anderen Risikogruppen*								
Almeida (2010) [2]	Brasilien	Programm für Epilepsiechirurgie	AB, Int	2,9^a^	16	550 MmE refraktär	1992–2002	k. A.
Annegers (2000) [4]	USA/weltweit	Epilepsiechirurgie	k. A.	4,1	13	1819 MmE VNS	1996–1999	k. A.
Donnan (2023) [12]	Australien	Populationsstudie	AB, Int, KA	2,51^a^	17	510 MmE mit DEE	k. A.	0–80 J
Esmaeili (2023) [16]	USA	Epilepsiezentrum	KA	0,88^a^	4	135 MmE OP LITT	2013–2021	16–80 J
Granbichler (2015) [19]	UK	Epilepsiezentrum/Epilepsiechirurgie	AB, KS, TS	3,3	10	466 MmE VNS	1995–2010	4–76 J
Jin (2002) [25]	China	Epilepsiemanagementprogramm	Int	2,47^a^	49	4296 MmE konvulsive Epilepsie	2012–2013	> 2 J
Hennessy (1999) [21]	UK	Epilepsiezentrum/Epilepsiechirurgie	AB, KA	2,2^a^	6	305 MmE OP	1975–1995	k. A.
Leestma (1997) [31]	USA	AED-Register	k. A.	3,13^a^	18	4700 MmE	k. A.	16–65 J
McKee (2000) [32]	USA	Einrichtung für Patienten mit geistigen Behinderungen	AB, KA, TS	3,6	11	180 MmE	1978–1997	k. A.
Nilsson (2003) [38]	Schweden	Epilepsiechirurgieregister	KA, Todesursachenregister	2,4	6	596 MmE OP	1990–1998	k. A.
Racoosin (2001) [43]	USA	AED-Klinische Studien	Fallberichte	3,8	52	9144 MmE	k. A.	k. A.
Rosenfeld (2023) [44]	USA	AED-Klinische Studien	KA	0,88	5	2132 MmE	k. A.	k. A.
Ryvlin (2018) [46]	USA	VNS-Therapie-Datenbank	AB, Int, KA	0,36^a^	101	40.443 MmE VNS	1988–2012	0–89 J
Seymour (2012) [52]	UK	Epilepsiezentrum/Epilepsiechirurgie	AB, KA, TS	1,68^a^	6	306 MmE OP	1975–2009	k. A.
Sillanpää (2016) [53]	Finnland	Epilepsiezentrum	TS	1,5^a^	10	214 MmE mit West Syndrom	1960–1976	k. A.
Sperling (2005) [55]	USA	Epilepsiezentrum	KA	3,63^a^	10	583 MmE OP	1986–2000	k. A.
Van der Lende (2018) [65]	Niederlande	Epilepsiezentrum	KA	3,53	60	k. A.	1987–2012	< 60 J

*AB* Autopsieberichte, *DEE* Entwicklungs- und epileptische Enzephalopathien, *Int* Interviews, *J* Jahre, *k.* *A.* keine Angaben, *KA* Krankenakte, *LITT* „laser interstitial thermal therapy“, *MmE* Menschen mit Epilepsie, *OP* operiert, *TS* Totenschein, *VNS* Vagusnervstimulation

^a^Die Werte wurden aufgrund von Angaben in der Publikation eigenständig berechnet

## Ergebnisse

Die systematische Recherche ergab insgesamt 3324 Publikationen, die anhand von Titel und Abstract unter Beachtung der prädefinierten Ein- und Ausschlusskriterien bewertet wurden. Abschließend wurden 41 Studien eingeschlossen. Zudem wurden die Referenzlisten bekannter Publikationen sowie systematischer Reviews nach weiteren potenziell relevanten Studien durchsucht. Diese Handsuche lieferte weitere 9 Studien.

### Merkmale der Studien

Die Studien wiesen eine hohe Heterogenität bei Kohorten und Studiendesigns auf. Die meisten Untersuchungen fanden in Europa statt, gefolgt von Nordamerika und dem asiatisch-pazifischen Raum. Datenquellen waren u. a. Krankenakten, Autopsieberichte, Patientenregister sowie Sekundärdaten (Tab. 2). Alle Studien nutzten zusätzliche Datenquellen wie Krankenakten zur SUDEP-Verifizierung. Durch unterschiedliche Datenquellen variierte die Methodik der Inzidenzberechnungen. So war z. b. bei 36 Studien die Basispopulation bekannt; andere schätzten die Anzahl der Epilepsiepatienten basierend auf einer Bevölkerungsprävalenz. Die Heterogenität der Kohorten spiegelt sich in dieser Arbeit wider. Es gab Studien nur mit Kindern und Jugendlichen [1, 20, 23, 28, 70], während andere diese Altersgruppe ausschlossen [26, 39, 69]. Die meisten Kohorten betrafen schwere/refraktäre Epilepsie (*n* = 17), Patienten in Epilepsiezentren (*n* = 13) und solche ohne spezifische Kriterien (*n* = 12) (Tab. 2). Zum Teil gab es überlappende Einschlusskriterien, so hatten Grønborg et al. [20] Kinder an einem Epilepsiezentrum untersucht. Um die Ergebnisse besser vergleichen zu können, wurden die Studien in die zuvor genannten Kategorien eingeteilt – ein Versuch, homogenere Populationen zu gewährleisten (Tab. 2).

### Inzidenz von SUDEP

Die größten Studien dieser Recherche stammen von Sveinsson et al. [60] und Tomson et al. [64] mit jeweils rund 60.000 Epilepsiepatienten. Als Registerstudien für die Gesamtbevölkerung bieten sie mit 0,78 bis 1,2 pro 1000 mitunter die repräsentativsten Inzidenzschätzungen. Studien, die die Inzidenz in der Gesamtbevölkerung messen, berichten von einer Spannweite von 0,33 bis 2,53, was ihre Heterogenität hervorhebt. Inzidenzen zwischen verschiedenen Altersgruppen lassen sich deshalb am besten innerhalb derselben Studien vergleichen. Die dänische Registerstudie von Kløvgaard et al. [30] berechnete eine Inzidenz von 0,27 für 1‑ bis 17-Jährige und 1,21 für 18- bis 49-Jährige. Als Kontrast dazu berichteten Keller et al. [27] ähnliche Inzidenzen für beide Altersgruppen, mit 0,79 für Personen < 16 Jahre und 1,1 für Personen > 16 Jahre. Für Kinder und Jugendliche ergibt sich in der Gesamtschau eine Spanne von 0,2 bis 1,11; für Erwachsene 0,72 bis 1,5. Studien an Epilepsiezentren oder Kliniken basieren auf spezifischen Gruppen und variieren im Studiendesign, was sich in einer Inzidenzspanne von 0,36 bis 7,1 zeigt. Ryvlin et al. [45] sticht mit 5,1 heraus und konzentriert sich auf Betroffene mit schweren Epilepsieformen, die in sog. Video-EEG-Monitoring-Einheiten gesehen werden. Sebera et al. [51] mit einer Inzidenz von 7,1 berücksichtigt nur wahrscheinliche Fälle, die durch Interviews mit Angehörigen bestätigt werden. Studien mit Kohorten aus Risikogruppen sind für eine allgemeine Inzidenzabschätzung ungeeignet, da das Studiendesign oft von spezifischen Fragestellungen beeinflusst wurde, wie etwa Nachuntersuchungen nach Operationen.

## Diskussion

Diese Arbeit resultiert aus einer systematischen Literaturrecherche und hat das Ziel, alle seit 25 Jahren nach Einführung einer einheitlichen Definition des plötzlichen unerwarteten Todes bei Epilepsie (SUDEP) relevanten Studien zur SUDEP-Inzidenz zu erfassen und zu analysieren. Zwei Übersichtsarbeiten zu diesem Thema wurden 2008 und 2018 veröffentlicht, beide mit ähnlichen Recherchekriterien. Saetre et al. [47] konzentrieren sich auf methodische Aspekte, während Tomson et al. [63] Studien nach Patientenpopulationen kategorisieren. Unsere Arbeit folgt dem Ansatz von Tomson et al. [63] und aktualisiert die 15 Jahre alte Veröffentlichung.

### Einflussfaktoren auf die SUDEP-Inzidenz

Die SUDEP-Inzidenz in den Studien zeigt starke Schwankungen, die durch das Studiendesign und die Auswahl der Kohorten bedingt sind. So weisen z. B. Epilepsiezentren – in denen der Anteil an Patienten mit schwer behandelbarer Epilepsie höher ist – tendenziell höhere SUDEP-Inzidenzen auf. Van der Lende et al. [65] verdeutlichen zudem, dass klinische Maßnahmen, wie nächtliche Supervision, die SUDEP-Inzidenz zwischen verschiedenen Zentren beeinflussen können. Die Studien wurden in fünf Gruppen unterteilt, um mehr Übersichtlichkeit zu schaffen, jedoch bleibt eine beachtliche Varianz innerhalb dieser Gruppen bestehen.

Ein weiterer Faktor, der die Vergleichbarkeit beeinflusst, ist die Heterogenität in der Datenerhebung. Die Daten stammen aus diversen Quellen, darunter Patientenregister, Fallstudien, Krankenakten und Autopsieberichte. Nicht zu vernachlässigen ist die Diagnose des SUDEP selbst: Sie basiert auf dem Ausschluss anderer Todesursachen statt auf eindeutigen Befunden. Besonders ohne Autopsie hängt die Diagnosegenauigkeit stark von der Methode der Datenerhebung ab [3, 36]. Ein weiterer wichtiger Einflussfaktor ist die Bestimmung der Größe der Epilepsiepopulation, welche zur Berechnung der Inzidenz verwendet wurde. So haben Keller et al. [28] in einer Sensitivitätsanalyse gezeigt, dass die Inzidenz bei Kindern zwischen 0,88 und 1,42 variieren kann, abhängig davon, welche Epilepsieprävalenz für die pädiatrische Epilepsie verwendet wurde.

### Patientengruppen im Fokus

Abseits der allgemeinen Datenerhebung bedürfen bestimmte Patientengruppen besonderer Aufmerksamkeit. So weisen einige Untersuchungen darauf hin, dass das SUDEP-Risiko insbesondere bei Kindern und Jugendlichen potenziell unterschätzt wird [30]. Dies wird durch die jüngsten Erkenntnisse von Borusiak et al. [6] untermauert, die darauf hinweisen, dass die verfügbare Datenlage zu SUDEP in dieser Altersgruppe immer noch dünn ist. Weitere Assoziationen zwischen dem Lebensalter und der Inzidenz ergeben sich aus Studien, die verschiedene Altersgruppen innerhalb derselben Kohorte betrachten. Fünf der analysierten Studien kategorisierten die Altersgruppen in 3 bis 4 Abschnitte. Nur Sveinsson et al. [60] verzeichneten einen durchgängigen Anstieg über alle Altersgruppen. Im Gegensatz dazu wiesen Einarsdottir et al. [14] den höchsten Wert für die 35- bis 54-Jährigen aus. Studien von Walczak et al. [68] und Clark et al. [10], die Altersdaten in 5‑ oder 10-Jahres-Intervallen analysierten, sahen einen Inzidenzanstieg bis zur Lebensmitte, der dann wieder abnahm. Ge et al. [19] erkannten bei Über-60-Jährigen keine SUDEP-Fälle. Ein Rückgang von (erkannten) SUDEP-Fällen im höheren Alter könnte durch mehr Komorbiditäten und sinkende Autopsieraten bedingt sein [11, 24, 60]. Uneindeutige Todesursachen erschweren die Klassifikation. Die Studien von Sveinsson et al. [60] als auch von Ge et al. [18] zeigen einen fortlaufenden Inzidenzanstieg in der höchsten Altersgruppe, wenn „mögliche SUDEP“-Fälle berücksichtigt werden.

Studien aus spezialisierten Epilepsiezentren zeigen höhere Inzidenzen, besonders bei refraktärer Epilepsie, einem bekannten SUDEP-Risikofaktor [33, 59]. Obwohl in 7 [4, 14, 18, 19, 42, 43, 60] von 10 [30, 68, 69] Studien Männer höhere Inzidenzen aufwiesen, ist der Geschlechtsunterschied nicht signifikant, sodass die Rolle des männlichen Geschlechts als SUDEP-Risikofaktor unklar bleibt [33, 59].

### Maßnahmen zur Prävention und Risikoreduktion

Unbeobachtete tonisch-klonische Anfälle sind ein Schlüsselfaktor für SUDEP [62]. Aufgrund des Zusammenhangs zwischen tonisch-klonischen Anfällen und SUDEP ist es plausibel, anzunehmen, dass eine bessere Anfallsüberwachung durch sog. Wearables das Risiko mindern kann [57]. Solche Technologien, die eine automatische Erkennung und Alarmierung ermöglichen, werden empfohlen [56], erfordern jedoch eine klare Kommunikation über SUDEP. Befürchtungen negativer Auswirkungen dieser Kommunikation auf die Lebensqualität von Patienten mit Epilepsie wurden widerlegt [57, 67].

Abnehmende SUDEP-Inzidenzen spiegeln positive Effekte durch erhöhtes Bewusstsein und besseres Management wider. Schulz et al. [50], Tomson et al. [64] und Wang et al. [69] verzeichneten deutliche Rückgänge nach Einführung eines Epilepsiemanagementprogramms. Verbesserte Aufklärung, Überwachung und Patientenmanagement werden in den jeweiligen Studien als mögliche Einflussfaktoren für den Inzidenzrückgang genannt.

### Limitationen und künftiger Forschungsbedarf

Die hohe Zahl der SUDEP-Fälle und die besondere Tragik durch den plötzlichen und unerwarteten Verlust betonen die Bedeutung des Themas und den Forschungsbedarf. Trotz gestiegenem Interesse erschweren methodologische Limitationen, wie Datenheterogenität und unterschiedliche Klassifikationen, z. B. Metaanalysen [22]. Künftige Forschung sollte Methoden standardisieren und mögliche Unterrepräsentationen prüfen. Ansätze zur Reduzierung der SUDEP-Inzidenz bis 2030 sind in Diskussion (www.SUDEP.de, www.SUDEP.org). Die Einbindung von SUDEP in die ICD-11 ab 2027 in Deutschland könnte die Datenerfassung vereinfachen.

## Fazit für die Praxis

SUDEP tritt bei 1 von 1000 Personen mit Epilepsie pro Jahr auf, meist als fatale Komplikation tonisch-klonischer Anfälle. Das individuelle Risiko ist unterschiedlich. Eine konsequente und vollständige Anfallskontrolle durch anfallssuppressive Medikamente kann das SUDEP-Risiko senken. Bei unzureichender Anfallskontrolle sollten früh weitere Therapieoptionen an einem spezialisierten Epilepsiezentrum geprüft werden. Alle Menschen mit Epilepsie und deren Angehörige sollen über das SUDEP-Risiko und Maßnahmen zur Risikoreduktion aufgeklärt werden, um ein aktives Management der Epilepsie und der Gestaltung individueller Lebensumstände zu ermöglichen. Der Einsatz von Wearables zur automatisierten Erkennung tonisch-klonischer Anfälle wird von diversen Fachgesellschaften empfohlen.
